# Artificial intelligence in cardiovascular imaging: state of the art and implications for the imaging cardiologist

**DOI:** 10.1007/s12471-019-01311-1

**Published:** 2019-08-09

**Authors:** K. R. Siegersma, T. Leiner, D. P. Chew, Y. Appelman, L. Hofstra, J. W. Verjans

**Affiliations:** 1 Department of Cardiology, location VUmc, Amsterdam University Medical Centres, Amsterdam, The Netherlands; 2 Department of Experimental Cardiology, University Medical Centre Utrecht, Utrecht University, Utrecht, The Netherlands; 3 Department of Radiology, University Medical Centre Utrecht, Utrecht University, Utrecht, The Netherlands; 4 Department of Cardiovascular Medicine, Flinders Medical Centre, Bedford Park, SA Australia; 5 South Australian Health and Medical Research Institute, Adelaide, SA Australia; 6 Cardiologie Centra Nederland, Amsterdam, The Netherlands; 7 Australian Institute for Machine Learning, University of Adelaide, Adelaide, SA Australia; 8 Dept of Cardiology, Royal Adelaide Hospital, Adelaide, SA Australia

**Keywords:** Artificial intelligence, Machine learning, Cardiac imaging techniques, Medical imaging, Clinical decision-making

## Abstract

Healthcare, conceivably more than any other area of human endeavour, has the greatest potential to be affected by artificial intelligence (AI). This potential has been shown by several reports that demonstrate equal or superhuman performance in medical tasks that aim to improve efficiency, diagnosis and prognosis. This review focuses on the state of the art of AI applications in cardiovascular imaging. It provides an overview of the current applications and studies performed, including the potential value, implications, limitations and future directions of AI in cardiovascular imaging.

It is envisioned that AI will dramatically change the way doctors practise medicine. In the short term, it will assist physicians with easy tasks, such as automating measurements, making predictions based on big data, and putting clinical findings into an evidence-based context. In the long term, AI will not only assist doctors, it has the potential to significantly improve access to health and well-being data for patients and their caretakers. This empowers patients. From a physician’s perspective, reliable AI assistance will be available to support clinical decision-making. Although cardiovascular studies implementing AI are increasing in number, the applications have only just started to penetrate contemporary clinical care.

## Introduction

Each year, more and more cardiac imaging investigations are being performed [52]. This is driven by multiple factors, such as increased acceptance of imaging, which over the years has played an incremental role in diagnosis, management and monitoring treatment outcome. In addition, imaging has become more widely available, and imaging equipment has become not only more precise, but also faster and cheaper. The improved quality and interpretability of imaging studies has not only led to increased satisfaction for the patient, but could also lead to increased reassurance of the doctor from a clinical and legal perspective. From an economical perspective, the global increase in healthcare costs is in part related to the increasing number of imaging units present in the hospital and thus the increased number of imaging studies performed [50]. However, the expansion of imaging capabilities and subsequent analyses stretches the limits of productivity of the average imaging specialist. Medical artificial intelligence (AI) is a solution for the standardised evaluation of the increasing number of medical images. Scientific literature has started to demonstrate that smart computers utilising AI can provide guidance and assistance during image acquisition and evaluation. This potentially has a significant influence on the physician’s workload.

### Why is AI promising for medical imaging?

One definition of AI is ‘the science of making machines do things that could be considered intelligent when they were performed by human beings’, although ‘intelligence’ itself could be considered a poorly defined term [41]. AI applications are increasingly used to solve problems in healthcare and medicine, as demonstrated by an increasing number of studies using keywords such as ‘artificial intelligence’, or the methodological references ‘machine learning’ (ML) and ‘deep learning’ (DL) [16]. The former refers to the development of models where input variables are predefined, e.g. the use of clinical, stress-testing and imaging variables for the prediction of major adverse cardiac events (MACE) [8]. The latter type of learning is based on the intrinsic discovery of important features in a multilayered model set-up, e.g. using echocardiographic images to classify the view [37].

AI researchers aim to develop and train self-learning models. These models pursue the identification of sophisticated relationships between a given input and corresponding outcome of multiple samples. As alluded to before, the definition of AI varies between experts, but they all refer to implementation of a distinctly human characteristic in models: exploiting previous experience to increase knowledge on how to perform a task in order to enhance decision-making in the future [55].

The notion of applying AI to medical imaging is fascinating for multiple reasons. First of all, it is becoming apparent that image datasets harbour considerably more useful data than a human can typically process. Secondly, simple tasks, like drawing contours and subsequent measurements, can be performed by computers more consistently, without interruption and many times faster than by humans. Although the development of useful ML models will take time, it is postulated that the implementation of AI will enable physicians to start working more efficiently [9, 15].

**Fig. 1:**
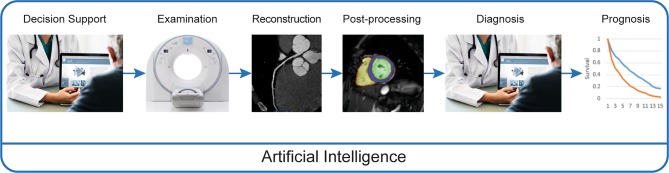
Artificial intelligence is able to impact all steps in the imaging chain

For medical imaging, AI impacts all steps of the imaging chain (Fig. 1). The first step is decision support for selection of the appropriate diagnostic imaging modality. Currently, healthcare is continuously pushing towards evidence-based decision-making and the use of guidelines. AI-based decision-support tools can aid in the selection of the most appropriate imaging test for individual patients. Furthermore, vendors are currently selling the first commercial products that implement ML during the examination of a patient [24, 37]. Following acquisition, AI is implemented in image reconstruction (e.g. using low-dose computed tomography, CT, to obtain an optimal anatomical reconstruction [72]), image interpretation and diagnosis (e.g. computer-aided diagnosis of myocardial infarction, MI, in echocardiography [60]). The final step in the imaging chain is to identify relevant prognostic and predictive information from cardiac imaging (e.g. prediction of adverse outcome in patients with pulmonary hypertension [17]).

The concept of personalised medicine is the combination of specific knowledge about an individual patient’s characteristics in order to tailor the predicted prognosis, choose treatment based on anticipated response or susceptibility for a specific disease [51]. Truly personalised medicine for multiple diseases is an important goal for the future of healthcare. For instance, direct application of AI would be very suitable for the evaluation of sex and gender differences, which is an important topic in current cardiovascular research. To accomplish this goal, different sets of data in healthcare must be combined: imaging data, electronic health records, biomarker analysis, genetic data, and others [54]. Although there have been attempts at combining different data sources, with some promising results [8, 44, 58], current research in AI has not yet reached this level of complexity in healthcare. Most published studies have focused on automated segmentation, post-processing and computer-aided diagnosis. Therefore, this review predominantly focuses on narrow AI projects that could prove useful in the near future in cardiovascular imaging. We aim to provide a non-systematic narrative overview of the early applications and studies of the implementation of AI in cardiac imaging, categorised by the different imaging modalities: echocardiography, CT, magnetic resonance imaging (MRI) and nuclear imaging.

## Implementation of AI in cardiovascular imaging

### Echocardiography

Echocardiography is the most widely used imaging modality in cardiology [26]. The advantages of ultrasound are portability, speed and affordability. However, it is a user-dependent method and intensive training is required in order to achieve accurate interpretation of the acquired data [21]. AI can aid in a more standardised analysis of echocardiographic images, to reduce user dependency. It has already demonstrated the ability to aid in the analysis of echo images, allowing the generation of important cardiac variables on-the-fly with automated classification of echocardiographic views (unpublished; DiA Imaging Analysis/GE Healthcare).

AI has been applied to different steps in the echocardiographic imaging chain. Firstly, during the acquisition of echocardiographic images, automated identification and measurement of the left ventricular wall has been implemented with an ML-based model. Performance of this algorithm is comparable to the traditional 3D echocardiographic methods and cardiac MRI. However, in a minority of pathologies, e.g. congenital disorders or disease with small ventricular cavities, the left ventricular myocardium is not optimally recognised by the implemented algorithm [64].

**Fig. 2:**
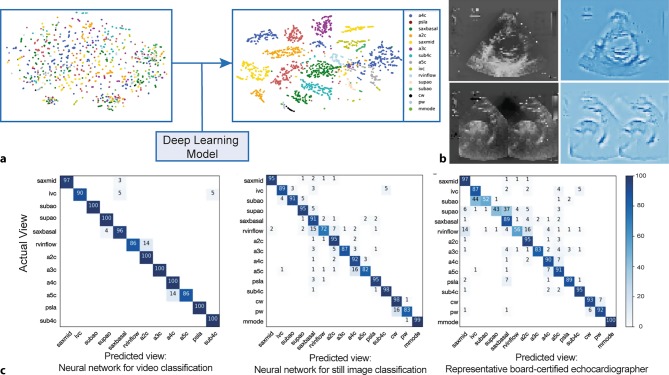
**a**–**c** Images obtained from the research performed by Madani et al. [37]. **a** 2D representation of the different echocardiographic views. Different colours represent the different standard echocardiographic views. A deep-learning model enabled classification, which resulted in the clustering as can be seen in the plot on the *right*. **b** The saliency maps (occlusion map not shown). The input pixels weighted most heavily in the neural network’s classification of the original images (*left*). The most important pixels (*right*) make an outline of relevant structures demonstrating similar patterns that humans use to classify the image. **c** The confusion matrices for different classifiers. The actual views are represented on the *vertical axes*. The *horizontal axes* represent the classification of views by a neural network with video classification input (*c1*), a neural network with still images as input (*c2*) and the classification performed by a board-certified echocardiographer (*c3*). The numbers in the squares represent the percentage of labels predicted for each category (rounding causes addition to not always add up to 100) [37]

Secondly, AI is applied in the post-processing of echocardiographic images. To facilitate a fully automated analysis, algorithmic classification of standard views is essential [31]. Madani et al. showed that a DL model achieves a similar performance in view classification to that of a board-certified echocardiographer (Fig. 2; [37]). Parameters of cardiac function have been analysed and determined with AI-based models trained with echocardiographic data. Results showed that the determination of left ventricular ejection fraction and longitudinal strain via AI generates similar results to those with expert visual determination [33]. Also, segmentation of the left and right ventricle is of interest, with the goal of automating ejection fraction measurements. The feasibility for the segmentation of the left ventricle using an AI model trained with small training sets has been demonstrated. The accuracy of the segmentation increased when the number of training images used was increased. This result shows an important characteristic of AI; increasing the amount of input data will usually improve the model’s performance. However, it should also be noted that using a more diverse dataset of images typically provides more generalisable results [13]. A similar performance was shown for determination of the size and function of the right ventricle. The correlation between automated and conventional right ventricular measurements ranged between 0.79 and 0.95 (*r*-values). However, Bland-Altman analysis showed that both end-diastolic and end-systolic volumes were usually overestimated in automated analyses. Furthermore, this method was semi-automated and required manual tracing of the right ventricular wall in a single frame [39]. All previously mentioned studies applied post-processing steps, performed after data acquisition. Excitingly, on-the-fly echocardiographic analysis has recently been introduced into the software of hand-held ultrasound devices, enabling automated analysis of variables during acquisition.

In addition to automated analysis, classifying or diagnosing several cardiac pathologies has been demonstrated. Moghaddasi and Nourian used three different classifiers for the detection of mitral regurgitation. A support vector machine provided the most accurate results for determination of severity (accuracy: >99% for every degree of severity), as evaluated by human interpretation [42]. Automated identification of MI has been enhanced with AI using different input features. Strain rate curves and segmental deformation for identification of MI demonstrated an accuracy of 87% [62]. Another study performed an analysis of MI using texture descriptors derived from the discrete wavelet transforms of the ultrasound signal (accuracy 99.5%) [60]. Narula et al. used speckle-tracking data to discriminate between an athlete’s heart and hypertrophic cardiomyopathy with three different classifiers. The models showed increased accuracy when different echocardiographic features were combined compared to single features alone. Although these pathologies are clinically similar, the ML model may present the opportunity to differentiate between phenotypes and modify therapy [47]. Similar echocardiographic data were used to differentiate between patients with restrictive cardiomyopathy and constrictive pericarditis. Although differentiation of these entities using four echocardiographic parameters without AI generates an area under the receiver operating characteristic curve (AUC) of 0.942, an associative memory classifier trained with features from speckle tracking echocardiography in addition to the four echocardiographic features generated an improved AUC of 0.962. While similar results were obtained with and without AI, this study demonstrates that implementation of AI for discrimination of these entities is feasible [57]. Zhang et al. published a study that includes segmentation, calculation of several clinical parameters and diagnosing three different cardiac pathologies in 14,035 echocardiograms. The application of AI was shown to be feasible in many steps along the imaging pathway, e.g. for detection of disease the AUC varied from 0.85 to 0.93 [73].

Another specific diagnostic domain for the implementation of AI is the characterisation of the phenotype of heart failure with preserved ejection fraction (HFpEF). This disease has a heterogeneous profile and its management is limited by the lack of a true gold standard definition [67]. In a study on 100 subjects, both HFpEF patients and healthy, but hypertensive and breathless, control subjects, a classifier had an accuracy of 81% for the classification of patients with HFpEF. This classification was based upon spatial-temporal rest-exercise features, which were partly determined by ML algorithms. This study shows the application of AI in diagnosis and post-processing of imaging, respectively [63]. Shah et al. also used a combination of imaging and clinical variables for classification and prediction of outcome in patients with HFpEF. This study showed an AUC between 0.70 and 0.76 during validation. Unsupervised phenomapping of HFpEF patients generated three different phenotypes with a significant difference in endpoints of cardiovascular hospitalisation or death. [58]. An unsupervised analysis of a combination of data sources has also been used in the identification of patients with heart failure that benefit from cardiac resynchronisation therapy [14].

In summary, in the short term AI will likely be implemented in echocardiography for automated segmentation and analysis of left and right ventricle contours and automated calculation of volumetric parameters, thereby reducing the workload of echocardiographic technicians. Subsequently, classification of disease with AI can be achieved, based solely on echocardiographic images, as well as combining imaging data with clinical variables, supporting clinicians and radiographers on the fly. This will also enable the generation of new hypotheses and lead to better diagnostic and prognostic performance in different cardiovascular pathologies.

### Computed tomography

Cardiac CT has made a leap forward in the last decade, focusing on the visualisation of stenosis in the coronary tree, plaque characteristics, coronary calcification and scoring and more recently the modelling of flow [49]. Promising opportunities for AI in CT are automated noise reduction, while retaining optimal imaging quality, and the avoidance of invasive coronary angiography (ICA) for determination of significant stenosis [11, 72].

In the context of image acquisition, Wolterink et al. described and validated a method with which to obtain reduced radiation dose CT images by training a DL model. Low-dose CT images were used to estimate the routine-dose CT images [72]. A similar approach with a convolutional neural network was used to determine the calcium score from regular coronary CT angiography (CTA). This obviated the need for calcium score CT and thereby reduced radiation exposure for the patient [70]. Another application of AI in CT is post-processing of the images. Zreik et al. showed that automated segmentation of the left ventricle from coronary CTA with convolutional neural networks is a feasible and reliable option [36].

**Fig. 3:**
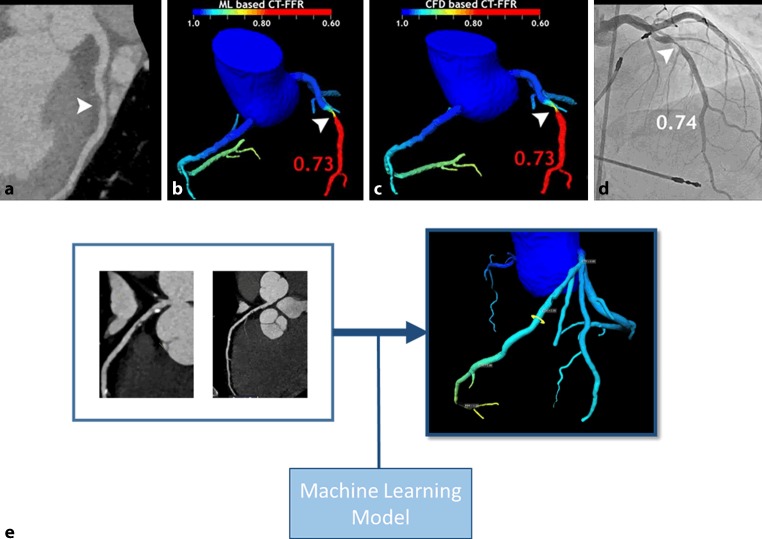
**a**–**e** A stenosis (indicated by the arrowheads) displayed in different imaging methods. **a** computed tomography coronary angiography (CTCA). **b** Calculation of fractional flow reserve (FFR) with a machine learning (*ML*) model. **c** Calculation of FFR with computational fluid dynamics (*CFD*). **d** Measurement of the stenosis during invasive coronary angiography. **e** From coronary CTCA to an AI-based 3D model of the coronary tree, displaying the FFR at different locations along the coronary arteries. (Images reproduced with permission [15])

A topic that has been extensively studied is the identification of significant coronary stenosis from coronary CTA. Significant coronary stenosis is defined as a fractional flow reserve (FFR) <0.8 determined during ICA. The use of AI replaces the need for invasive measurements and generates clear models of local FFR (Fig. 3). Different input features derived from coronary CTA have been used for modelling: physiological features [28], quantitative plaque measurements [18], features calculated from different spatially connected clusters of heart segmentation [75], and geometric features of the coronary anatomy [15, 66]. Also features from CT perfusion are being evaluated for use with AI [25]. Currently, non-invasive measurements of FFR are performed with computational fluid dynamics, which is computationally demanding. Substitution of this method by AI was shown to be faster and performance was equally good [15, 28]. Improvement of non-invasive determination of FFR was obtained by accounting for partial volume effects with AI. Partial volume effects lead to an overestimation of the vessel lumen area [23]. This development leads to an opportunity to decrease the number of ICAs, while allowing for targeting specific stenosis during ICA and, thus, decreasing the duration of the procedure. Automated identification of coronary artery calcium (CAC) has also been subjected to AI approaches, showing that automated identification of CAC in ECG-gated non-contrast-enhanced CT imaging has an intra-class correlation coefficient of 0.95. This performance is similar to that of a human expert [71].

A small number of studies have been performed to determine the diagnostic value of AI in coronary CTCA. Zreik et al. obtained an accuracy of 0.77 for the detection and characterisation of coronary plaque. For the detection of stenosis and determination of the anatomical significance, an even higher accuracy of 0.80 was obtained, with a dataset of 163 patients [76]. Kolossváry et al. used a more supervised approach with predefined measurements, so-called radiomics [1] features, to identify coronary plaques with a napkin-ring sign (NRS). This sign is an independent prognostic marker of MACE. A large number of texture features, derived from the radiomics set, are able to differentiate between plaques with and without NRS [34]. Another study used texture features derived from calcium score CT as the input in ML models to discriminate between patients with acute or chronic MI and control subjects. This resulted in an AUC of 0.78 and obviated the need for gadolinium-enhanced MRI [38]. However, it has to be noted that manual segmentation of the coronary plaques and the left ventricular wall was required, creating an extra non-automated action. This limits the possible implementation in the cardiologist’s clinical workflow.

Besides automated analysis and diagnostics, prognostic evaluation has been applied in cardiac CT. Survival analysis was performed in different patient groups with a cardiovascular risk. In the classification of all-cause mortality among patients with suspected coronary artery disease (CAD), ML models exhibited a larger AUC (0.79) than the individual clinical and coronary CTCA metrics (e.g. Framingham risk score: 0.61, segment stenosis score: 0.64, segment involvement score: 0.64, Duke index: 0.62) [44]. A similar prognostic model was developed using coronary CTCA features derived from the stenosis. This model generated a risk score for all-cause death and non-fatal MI during a follow-up of >3 years and resulted in an AUC of 0.771. This AUC was higher than for each of the individual conventional coronary CTCA variables [53].

Results of AI-based models are promising for cardiac CT; more specifically there is a great future for coronary CTCA, mainly due to the non-invasive nature of CT imaging, which is relatively user-independent and fast. Reducing the radiation dose is a relevant application of AI for patients, but preserving spatial resolution is important to make appropriate diagnostic and possibly prognostic decisions. Another purpose of AI in coronary CTCA is reducing the need for ICA by expanding the informational value of the diagnostic images. Given the number of studies that apply AI in CT, this field is expanding and starting to incorporate other data sources in the analysis. This creates valuable models and brings us closer to a world of personalised medicine.

### Magnetic resonance imaging

Cardiac MRI is a field that comprises the imaging of many aspects of the heart: anatomical imaging, contractile function, flow imaging, perfusion imaging and, importantly, myocardial characterisation [22]. However, given the many opportunities that cardiac MRI offers with regard to AI applications and the technological methods used in MRI, radiographers that have experience and knowledge of physics and cardiac anatomy are integral to image acquisition and analysis. As a consequence, the quality of cardiac MR images is not only user dependent, but also patient, scanner and vendor dependent.

**Fig. 4:**
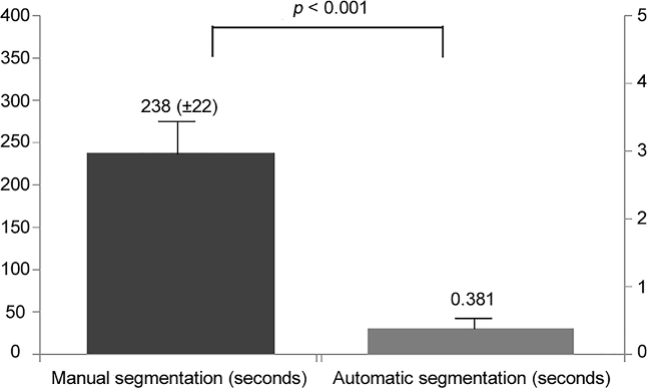
Comparison of processing times of segmentation of the aortic valve in cardiovascular magnetic resonance phase contrast imaging. Automated segmentation used a neural network approach, trained with 150 segmentations. Validation was done in a cohort of 190 segmentations. Automated segmentation times were obtained with GPU acceleration. However, also without GPU acceleration, the average segmentation time was 19.04 s. (Images obtained from Bratt et al. [10])

Automated segmentation of cardiac structures and infarct tissue have been the main topic of interest in cardiac MRI thus far. Several studies have been published on the automated segmentation of cine images [5, 10, 48, 65, 74] and automated calculation of cardiac parameters from MRI [7, 61]. Multiple software programs are available that perform automated segmentation based upon AI. Algorithms for automated segmentation of enhancement on late gadolinium enhancement imaging were summarised and tested by Karim et al., whose study showed that AI algorithms provided greater accuracy than fixed-model approaches [30]. Beyond performance, the reduction in time is a particularly important characteristic of automated AI-based segmentation, as can be seen in Fig. 4; [10]. Baessler et al. used ML models to select the most important texture features, derived from cine images, to differentiate between patients with MI and control subjects. The use of two texture features in multiple logistic regression generated an AUC of 0.92 [6]. Implementation of this model in a clinical setting precludes the need for gadolinium-enhanced cardiac MRI, potentially expanding the eligible patient population and reducing costs. All these studies suggest the feasibility of simplifying further analysis of myocardial tissue in large cardiac MRI datasets. An interesting approach by Snaauw et al. demonstrated to possiblity of so-called end-to-end classifcation of disease on cardiac MR images, without the need for annotation [59].

Two studies have been reported that perform predictive modelling with cardiac MRI data. First, principal component analysis was used to determine survival in patients with pulmonary hypertension. Input for the analysis was the three-dimensional cardiac motion of the right ventricle. This method showed an AUC of a time-dependent receiver operating characteristic analysis of 0.73 for the inclusion of 3D-MR features in the model, besides clinical, functional and regular MR features and features derived from right-sided heart catheterisation (otherwise: AUC 0.60). Median follow-up time was 4.0 years [17]. A second predictive model examined the deterioration of left ventricular function in patients with a repaired tetralogy of Fallot. This study indicated that ML models can be useful for planning early intervention in patients at high risk (AUC: 0.87 for major deterioration). Follow-up duration had a median of 2.7 years [56].

To conclude, due to major disadvantages that compromise inter- and intra-patient comparability in MRI, the application of diagnostic and prognostic AI in MRI is more challenging than in other imaging modalities. In the short term, the use of AI in cardiac MRI will therefore be primarily focusing on automated segmentation and calculation of variables. There are vendors who have incorporated this into their software. Nonetheless, efforts to increase the use of AI for diagnostics and prognostics in CMR continue, with the key challenge of overcoming the between-study comparability of MR images. Methods are being developed and currently further optimised and standardised.

### Nuclear imaging

Nuclear imaging of the heart is used to assess perfusion defects within the myocardial wall. Myocardial perfusion single-photon emission computed tomography (SPECT) and positron emission tomography (PET) are methods for cardiac nuclear imaging, although the latter is rarely used in clinical practice due to its costliness, among other reasons. Whereas PET is based on the simultaneous detection of two opposite annihilation photons, SPECT uses gamma rays emitted by a radioactive tracer to reconstruct tissue with uptake. Both nuclear imaging methods can be combined with MRI or CT, which has shown to improve their clinical value, although cardiac PET-MRI has only just started to be used in larger centres [12, 32].

The automated analysis of SPECT is a growing field of interest for research. Normal and abnormal myocardium in CAD can be classified with AI-based models, with performance reported to be similar to the visual analysis of SPECT images [19]. Also, the detection of locations with abnormal myocardium has been investigated. An artificial neural network (ANN), trained with expert interpretations of SPECT images, improved the identification of stress (AUC: 0.92), rest defects (AUC: 0.97) and stress-induced ischaemia (AUC: 0.97) compared to conventional scoring; the AUC of the summed stress score, summed difference score and the summed rest score was 0.82, 0.75 and 0.91, respectively [45]. Another study by this group compared an improved version of the ANN to the older version, showing that retraining of the model improved the identification of ischaemia. The AUC increased to 0.96 [46].

The accuracy of SPECT can be boosted by the integration of clinical data and quantitative imaging features in an ML model. The diagnostic accuracy in the detection of obstructive CAD was improved with an ML model with quantitative and clinical features. This model generated a marginally better result than a model with solely clinical features (accuracy: 79.4% vs 75.7%). The performance of the model was similar to the visual analysis of one experienced reader (78.5%) and better than another (73.5%).[3]. Also, Betancur et al. examined the automated prediction of obstructive CAD. DL models were trained with the raw and quantitative perfusion polar maps. AI-based models showed a higher AUC (0.80) for prediction of CAD than the current clinical method (0.78) in 1638 subjects.[9]. Another study showed utility in aiding decision-making for cardiac interventions. SPECT data, merged with functional and clinical features, were used to predict the necessity for revascularisation. The results of this study showed that an ML approach (AUC: 0.81) was comparable to or better than two experienced readers (AUC: 0.81 and 0.72) in the prediction of the need for revascularisation [4].

MACE were also studied in 2619 patients who were referred for myocardial perfusion imaging. This risk analysis was based upon an ML model that combined clinical information with myocardial perfusion SPECT data. This model showed a higher AUC than a model with solely imaging features (AUC: 0.81 vs 0.78) [8]. Another study by Haro Alonso et al. compared an ML model with baseline logistic regression (LR) for the prediction of cardiac death. Patients were selected if they had undergone myocardial perfusion SPECT and imaging parameters were used for modelling. The study showed that baseline LR (AUC: 0.77) was outperformed by all ML models, with the support vector machine generating the highest AUC (0.83) [2]. ML models have also been used in cardiac PET. However, in this case PET variables were used as the output classification, using demographic, clinical and functional variables as input. ML models were superior to LR for the identification of myocardial ischaemia, based upon PET images, and selection of patients at risk for MACE [29].

To conclude, short-term applications for AI in nuclear imaging are predominantly focused on automated detection of perfusion defects in the myocardial wall. Because nuclear imaging can be easily combined with CT or MRI, this enables enhanced fusion of multiple data sources in addition to clinical data. Such methods have been shown to improve the performance of diagnostic and predictive models. However, in the long term, the high radiation exposure during nuclear imaging remains a limitation in the cardiac clinic, and hence also to the penetration of AI into this imaging modality.

## Conclusions and future directions

Cardiovascular imaging has shown remarkable advancements in the last few decades, leading to detailed imaging of not only structural, but also physiological and even molecular characteristics of the cardiovascular system. The advancement of AI creates opportunities in healthcare to obtain more sophisticated information from imaging, and to find patterns in available data sources that are too complex for the human brain. Intuitively, it is more a question of when, rather than if, AI technology will offer significant help for the cardiologist, and in particular the imaging specialist. Applications are already being implemented in the clinical workflow based on research that shows equal or better performance than analysis by the physician or conventional (semi-)automated methods, with the aim of reducing the workload of the physician and enhancing decision-making.

The use of AI in cardiac CT applies to many steps of the imaging chain, whereas the application of AI in cardiac MRI has so far primarily focused on automated segmentation of anatomical structures of the heart. In addition, nuclear imaging and echocardiography have used predictive and prognostic modelling. Nevertheless, the implementation of AI faces significant challenges. There are efforts underway to improve the comparability of imaging modes. Automated segmentation or extraction of imaging features is likely to be ‘solved’ first, and this will help to standardise and accelerate the analysis of large datasets.

Despite promising results, the implementation of AI in contemporary cardiovascular healthcare has been limited to date [27, 40]. Several reasons contribute to this observation. First, regulatory bodies, like the American Food and Drug Administration, have difficulty with the regulation and approval of software based on AI [68]. This delays the allotting of certification marks and the introduction of products on the consumer market. Second, the added value of AI in clinical care remains to be determined and established. No studies have been performed that show that the implementation of AI indeed leads to higher quality of care, lower healthcare costs or improved patient outcomes [54]. Furthermore, due to the ‘black box’ used in many ML models and the dependency on input data for the performance of a model, it is difficult to replicate or explain experiments. Repeating studies and validating designed ML models will be important before routine implementation in clinical practice [20]. This requires sharing of data and model settings, which is uncommon in clinical research; also patient privacy and compliance with regulations regarding patient data are critical considerations. Third, physicians are not yet prepared for the implementation of AI in the daily clinical setting. Trust in these new technologies has to be built, supported by efforts towards transparency and explainability [35]. Fourth, the datasets used in the described studies are commonly relatively small. A large range of different patients must be included in studies to develop appropriate models. Diversity in ethnicity, gender and age must be guaranteed to build widely applicable models. This includes the data used during training, validation and testing of the model. Furthermore, a standardised method for storing or extraction of information in the electronic health record should be developed. The use of free text should be reduced to enhance data analysis and application of AI.

There is also an opportunity for future research to focus on the implementation of data from multiple sources in ML models, including biomarkers, genomics, proteomics and metabolomics [54]. This can improve predictive value of ML models and create personalised healthcare for patients. Text mining and improvement of the predictive value of free text analysis are being explored [43, 69], but standardised reporting can clearly facilitate the implementation of AI worldwide. The implementation of multiple sources in ML models can also contribute in deciding whether to refer a patient for cardiac imaging, e.g. immediate therapeutic decision-making based on CT data without the need for ICA [66].

Current AI technology is considered to be ‘narrow’, meaning it is good at one particular task, and it is only as good as the dataset that trained it. AI has made remarkable progress, and despite a clear peak of potentially inflated expectations, the number of translational studies that implement so-called narrow AI is slowly growing, with some results already showing performance that is equal to or better than that of conventional methods (e.g. [44]) or expert analysis (e.g. [3]). It will take more than a few decades before we are able to achieve so-called general, human-like AI. It will undoubtedly take time for the adoption of such methods in daily clinical practice, where decisions are complex. Moreover practice is relatively conservative in the face of ethical and medicolegal considerations [35].

Physicians need to realise that AI is a tool that will not replace many tasks in the short term, but will likely enhance diagnostic and decision-making capacity. Human performance will be augmented, and it is likely this will improve the outcome of patients through better diagnosis, fewer errors and significant time-saving that could help us create more productive patient-doctor interactions.
